# Interaction between prenatal pesticide exposure and a common polymorphism in the *PON1* gene on DNA methylation in genes associated with cardio-metabolic disease risk—an exploratory study

**DOI:** 10.1186/s13148-017-0336-4

**Published:** 2017-04-05

**Authors:** Ken Declerck, Sylvie Remy, Christine Wohlfahrt-Veje, Katharina M. Main, Guy Van Camp, Greet Schoeters, Wim Vanden Berghe, Helle R. Andersen

**Affiliations:** 1 Laboratory of Protein Chemistry, Proteomics and Epigenetic Signalling (PPES), Department of Biomedical Sciences, University of Antwerp, Universiteitsplein 1, Antwerp, Belgium; 2 Department of Epidemiology and Social Medicine, Antwerp University, Universiteitsplein 1, Antwerp, Belgium; 3 Flemish Institute for Technological Research (VITO), Unit Environmental Risk and Health, Boeretang 200, Mol, Belgium; 4 Department of Growth and Reproduction, University Hospital of Copenhagen, Rigshospitalet, Copenhagen, Denmark; 5 Center of Medical Genetics, University of Antwerp and Antwerp University Hospital, Antwerp, Belgium; 6 Department of Biomedical Sciences, Antwerp University, Universiteitsplein 1, Antwerp, Belgium; 7 Environmental Medicine, Institute of Public Health, University of Southern Denmark, Odense, Denmark

**Keywords:** DNA methylation, Prenatal pesticide exposure, Paraoxonase 1, *PON1* Q192R genotype, Illumina 450 K methylation array, Cardio-metabolic health

## Abstract

**Background:**

Prenatal environmental conditions may influence disease risk in later life. We previously found a gene-environment interaction between the paraoxonase 1 (*PON1*) Q192R genotype and prenatal pesticide exposure leading to an adverse cardio-metabolic risk profile at school age. However, the molecular mechanisms involved have not yet been resolved. It was hypothesized that epigenetics might be involved. The aim of the present study was therefore to investigate whether DNA methylation patterns in blood cells were related to prenatal pesticide exposure level, *PON1* Q192R genotype, and associated metabolic effects observed in the children.

**Methods:**

Whole blood DNA methylation patterns in 48 children (6–11 years of age), whose mothers were occupationally unexposed or exposed to pesticides early in pregnancy, were determined by Illumina 450 K methylation arrays.

**Results:**

A specific methylation profile was observed in prenatally pesticide exposed children carrying the *PON1* 192R-allele. Differentially methylated genes were enriched in several neuroendocrine signaling pathways including dopamine-DARPP32 feedback (appetite, reward pathways), corticotrophin releasing hormone signaling, nNOS, neuregulin signaling, mTOR signaling, and type II diabetes mellitus signaling. Furthermore, we were able to identify possible candidate genes which mediated the associations between pesticide exposure and increased leptin level, body fat percentage, and difference in BMI *Z* score between birth and school age.

**Conclusions:**

DNA methylation may be an underlying mechanism explaining an adverse cardio-metabolic health profile in children carrying the *PON1* 192R-allele and prenatally exposed to pesticides.

**Electronic supplementary material:**

The online version of this article (doi:10.1186/s13148-017-0336-4) contains supplementary material, which is available to authorized users.

## Background

A considerable part of modern pesticides has neurotoxic and/or endocrine disrupting properties [1–3] and therefore the potential to disturb development of neurobehavioral, neuroendocrine, and reproductive functions [4–8] especially if exposure occurs during vulnerable time periods in fetal life or early childhood. To investigate potential health effects of prenatal pesticide exposure, we have followed a cohort of children, whose mothers were employed in greenhouse horticulture in pregnancy. Some of the mothers were occupationally exposed to mixtures of pesticides in the first trimester before the pregnancy was recognized, and preventive measures were taken. Findings from this cohort include associations between maternal pesticide exposure and lower birth weight followed by increased body fat accumulation during childhood [9], impaired reproductive development in boys [10, 11], and earlier breast development [12] and impaired neurobehavioral function in girls [13].

The HDL-associated enzyme paraoxonase 1 (PON1) catalyzes the hydrolysis of a wide range of substrates including some organophosphate insecticides [14, 15]. It also protects lipoproteins from oxidative modifications and hence against development of atherosclerosis [16, 17]. A common polymorphism in the coding sequence of the *PON1* gene substitutes glutamine (Q) to arginine (R) at position 192. This substitution seems to affect both properties of the enzyme, and several studies have indicated an increased risk of cardiovascular disease in R-allele carriers [17, 18]. To investigate if this polymorphism affected the sensitivity to prenatal pesticide exposure, the *PON1* Q192R genotype was determined in the children. We found a marked interaction between prenatal pesticide exposure and the *PON1* Q192R genotype. At school age, exposed children with the R-allele had significantly higher BMI, body fat percentage, abdominal circumference, and blood pressure compared to unexposed children with the same genotype. In the group of children with the QQ genotype, there was no effect of prenatal pesticide exposure on these parameters [19]. In addition, serum concentrations of leptin, glucagon, and plasminogen activator inhibitor type-1 (PAI-1) were enhanced in prenatally pesticide exposed children with the R-allele, also after adjusting for BMI [20] which also indicates disturbance of metabolic pathways related to development of metabolic syndrome [21–23]. In addition, leptin seemed to be a mediator of the increased fat accumulation during childhood related to prenatal pesticide exposure in children with the *PON1* 192R-allele [20]. Thus, the obtained results indicate a gene-environment interaction between pesticide exposure and *PON1* gene heterogeneities already in early prenatal life that might enhance the risk of cardio-metabolic diseases later in life.

The mechanism behind this interaction is not yet understood but might be mediated by epigenetic alterations depending on both genotype and prenatal exposure. Epigenetic marks, including DNA methylation and covalent histone modifications, are dynamic and can adapt to a variety of external stimuli [24]. Furthermore, during fetal development extensive de- and re-methylation events are taking place making this period highly vulnerable for epigenetic changes caused by environmental conditions [25]. Indeed, emerging evidence in experimental animals and in humans associate altered DNA methylation patterns with a variety of prenatal exposures including dietary factors, parental care, infections, smoking, and environmental pollutants [26–31]. In experimental animals, early life changes in DNA methylation have been associated with diet-induced obesity and insulin resistance [32]. Recently, also human studies have suggested that DNA methylation patterns at birth are related to birth weight and fat mass later in childhood [33, 34]. The aim of this exploratory study was to investigate whether methylation patterns in blood samples of school children were related to prenatal pesticide exposure, *PON1* Q192R genotype, and adverse health outcomes already observed in the children. We hypothesized that the health effects associated with early prenatal pesticide exposure were related to differential epigenetic modifications in children with the QQ-genotype and children carrying the R-allele.

## Methods

### Study population

This study is a part of an ongoing prospective study including 203 children born between 1996 and 2001 by female greenhouse workers. The children were examined for the first time at 3 months of age [11] and followed-up at school age when 44 new age-matched controls were included [9], and the *PON1* genotype was determined for 141 children [19]. For this exploratory study, 48 pre-pubertal (Tanner Stage 1) children, whose mothers reported not to have smoked during pregnancy, were selected equally distributed between the *PON1* 192QQ and QR/RR genotype. The QR/RR genotype group consisted of 3 children with the RR genotype and 21 with the QR genotype. After excluding children of mothers who smoked in pregnancy, the number of unexposed controls within each genotype was low, 20 with the QQ genotype and 16 with the QR/RR genotype. DNA qualified for methylation analysis was only available for 11 and 12 of these children, respectively. For each genotype, we then used individual matching to select one exposed child of same sex and age for each of the controls. For the QQ-genotype, two exposed children were selected for each of two controls to obtain 24 children. Thus, in total we used data from 13 exposed and 11 unexposed children with the QQ genotype, and 12 exposed and 12 unexposed children with the QR/RR genotype (Table 1).

**Table 1:** Population characteristics and anthropometric data for 48 pre-pubertal children examined at age 6–11 years stratified by *PON1* Q192R genotype and prenatal pesticide exposure

	*PON1* 192QQ		*PON1* QR/RR	
	Unexposed	Exposed	Unexposed	Exposed
*N*	11	13	12	12
Female sex	5 (45.5)	7 (53.8)	6 (50.0)	6 (50.0)
Maternal smoking in pregnancy	0 (0)	0 (0)	0 (0)	0 (0)
SES^a^	7/4 (63.6/36.4)	3/10 (23.1/76.9)*	5/7 (41.7/58.3)	2/10 (16.7/83.3)
Birth weight (g)	3640 (2600; 5412)	3382 (2750; 4573)	3789 (2984; 4345)	3500 (2900; 3914)*
Gestational age (days)	276 (257; 291)	283 (265; 295)	283 (261; 298)	281 (266; 291)
Age (years)	7.6 (6.2; 9.8)	8.4 (6.7; 10,0)	7.8 (6.6; 9.5)	7.7 (7.1; 9.4)
Height (cm)	133.3 (117.3; 145.2)	130.3 (109.7; 139.2)	130.9 (113.7; 149.1)	128.6 (119.3; 142,5)
Weight (kg)	30.9 (18.7; 38.0)	28.3 (18.0; 30.7)	26.3 (19.9; 36.5)	27.4 (19.5; 37.8)
BMI (kg/m^2^)	16.2 (13.7; 20.5)	15.3 (14.9; 18.3)	15.5 (13.8; 16.9)	15.7 (13.8; 19.7)
BMI *Z* scores	0.66 (−1.03; 3.21)	−0.18 (−0.80; 1.49)	−0.04 (−1.31; 0.89)	−0.01 (−0.98; 3.14)
Delta BMI *Z* score since birth	−0.45 (−2.15; 2.97)	−0.71 (−2.57; 1.87)	−0.56 (−2.52; 1.03)	0.95 (−2.08; 2.97)*
Abdominal circumference (cm)	60.4 (52.0; 75.8)	58.7 (52.1; 66.8)	58.3 (52.0; 68.1)	60.8 (51.8; 70.6)*
Sum of four skin folds (mm)	38.4 (27.1; 85.4)	33.6 (25.4; 54.5)	34.0 (20.2; 45.2)	44.6 (28.8; 72.0)*
Systolic blood pressure (mmHg)	98.7 (93.7; 110.4)	97.2 (84.3; 105.3)	99.7 (84.7; 106.8)	101.7 (91.0; 108.6)
Diastolic blood pressure (mmHg)	54.7 (46.0; 69.9)	56.2 (46.0; 62.0)	56.3 (49.3; 69.1)	63.0 (57.3; 73.1)**
Leptin (ng/ml)	1.47 (0.70; 9.18)	4.40 (0.60; 15.29)	1.41 (0.67; 5.90)	4.69 (1.79; 12.25)**
Insulin (ng/ml)	0.36 (0.22; 1.15)	0.52 (0.23; 2.55)	0.34 (0.16; 1.62)	1.11 (0.24; 7.10)*
Paraoxonase activity (nmol/min/ml)	27.5 (9.9; 38.0)	30.9 (21.0; 38.9)	58.6 (41.9; 68.7)	59.6 (50.3; 71.5)

^a^ *SES* socioeconomic status (social class 1–3/4–5). Differences between unexposed and exposed children for each *PON1* Q192R genotype were tested using Mann-Whitney *U* test for continuous variables and Fisher’s exact test (dichotomous variables) or Likelihood ratio (categorical variables with > 2 categories). *P value ≤ 0.05, **P value ≤ 0.01. Values are presented as median (5–95%) for continuous variables and as *N* (%) for categorical variables

Recruitment, characteristics, exposure categorization, and clinical examinations of the children have previously been described in detail [9, 11, 19]. Briefly, we recruited pregnant women working in greenhouses and referred to the local Department of Occupational Health for risk assessment of their working conditions and guidance for safe work practices during pregnancy. Detailed information about working conditions inclusive pesticide use for the previous 3 months was obtained from maternal interview at enrollment (gestational weeks 4–10) and supplemented by telephone contact to the employers. For all women, re-entry activities (such as moving or packing potted plants or nipping cuttings) constituted their main work functions. Approximately 20% of the women reported having been directly involved in applying pesticides, mainly by irrigating fungicides or growth retardants. Only few (6%) of the women had applied insecticides. The women were categorized as occupationally exposed if pesticides were applied in the working area more than once a month, and the women handled treated plants within 1 week after treatment and/or the women were directly involved in applying pesticides. The women were categorized as occupationally unexposed if none of the above criteria was fulfilled. All exposure assessments and categorization of the mothers as pesticide exposed or unexposed were performed independently by two toxicologists before the first examination of the children. Women categorized as pesticide exposed went on paid leave or were moved to work functions with less or no pesticide exposure shortly after enrollment. Hence, the exposure classification relates to the early weeks of the first trimester before study enrollment.

The exposure situation was complex since the use of specific pesticides varied with time and location, both within the same company and between companies, depending on the plant production and the type of pest to be controlled. Out of 124 different active pesticide ingredients used in the greenhouses were 59 insecticides (17 organophosphates, 12 pyrethroids, 9 carbamates, and 21 others), 40 fungicides, 11 growth regulators, and 14 herbicides. Some were used only in few greenhouses or in short periods, whereas others were used more often. Organophosphate insecticides were used to some extent in the working areas for 91% of the exposed mothers in the entire cohort, and for 24 out of the 25 exposed mothers whose children were included in this study. The most used organophosphates were dichlorvos, dimethoate, and chlorpyrifos. Other frequently used pesticides were the pyrethroid insecticides deltamethrin and fenpropathrin; the carbamate insecticides methiocarb, pirimicarb, and methomyl, and the fungicides fenarimol, prochloraz, tolclofos-methyl, vinclozolin, iprodion, and chlorothalonil. In general, the time interval between applying insecticides and working in the treated areas was longer (1–3 days) than for fungicides and growth regulators (often a few hours). Because of the complexity of the exposure situation and because most of the women at enrollment had been off work for some days while the risk assessment of their working conditions was performed, biomonitoring of the exposure was not feasible. A complete list of the pesticides used in the greenhouses can be obtained from the corresponding author.

At follow-up at age 6 to 11 years, 177 children underwent a standardized clinical examination in which systolic and diastolic blood pressure, pubertal staging, height, weight, thickness of skin folds, and other anthropometric parameters were measured [9]. The same pediatrician performed all clinical examinations blinded to information about maternal pesticide exposure during pregnancy.

Venous non-fasting blood samples were collected (between midmorning and late afternoon) in EDTA-coated and uncoated vials (Venoject). After centrifugation at 2000 *g* for 10 min at 20 °C, buffy coat for genotyping and epigenetic analysis was separated from the EDTA-treated samples. Buffy coat and serum from the uncoated vials were stored at −80 °C until analysis.

As previously described [19], C-108T (rs705379) and Q192R (rs662) polymorphisms of the *PON1* gene was determined by the Taqman-based allele discrimination using the ABI Prism 7700 Sequence Detection System, serum activity of *PON1* was determined by spectrophotometry with paraoxon as substrate, and insulin (proinsulin and insulin) and leptin concentrations in serum were determined by commercial ELISA hormone kits from RayBio.

Genotyping and all serum analyses were performed blinded to both exposure information and examination outcomes.

### Sample preparation

DNA from buffy coat samples was extracted using QIAamp DNA Blood Mini Kit (Qiagen, Hilden, Germany). The blood spin protocol was applied according to manufacturer’s instructions. Samples were eluted in 100 μl elution buffer. DNA samples were bisulfite-converted using the EZ DNA methylation kit from Zymo according to manufacturer’s instructions. Successful bisulfite conversion was checked using a bisulfite-specific PCR of an amplicon in the *SALL3* gene (see Additional file 1 for primer sequences). Only samples showing an intense band on agarose gel were further analyzed by the 450 K methylation array. As a negative control non-converted gDNA was used.

### DNA methylation and data preprocessing

The Infinium HumanMethylation450 BeadChip array (Illumina, San Diego, CA, USA) was used to measure DNA methylation genome-wide. 4 μL of bisulfite-converted DNA from each sample was amplified, fragmented, precipitated, resuspended, and subsequently hybridized onto the BeadChips. After overnight incubation of the BeadChips, unhybridized fragments were washed away, while hybridized fragments were extended using fluorescent nucleotide bases. Finally, the BeadChips were scanned using the Illumina iScan system to obtain raw methylation intensities for each probe.

We used the R package RnBeads to preprocess the Illumina 450 K methylation data [35]. Cg-probes were filtered before normalization based on following criteria: probes containing a SNP within 3 bp of the analyzed CpG site, bad quality probes based on an iterative Greedycut algorithm where a detection *p* value of 0.01 was set as a threshold for an unreliable measurement, and probes with missing values in at least one sample. After filtering these cg-probes, beta values (ratio of methylated probe intensity versus total probe intensity) were within-array normalized using the beta mixture quantile dilation (BMIQ) method [36]. Another filtering step was performed after normalization based on the following criteria: probes measuring methylation not at CpG sites and probes on sex chromosomes. The two filtering steps removed a total of 20,338 cg-probes and ended up with a data set of normalized methylation values for 465,239 cg-probes. Beta values were transformed to *M* values (*M* = log_2_(β/(1−β))) prior to further analyses. Principal component analysis (PCA) was conducted to detect possible batch effects. Associations between the first eight principal components and possible batch effect covariates were measured. The Kruskall-Wallis test was used to find associations with sentrix_ID (BeadChip), while the two-sided Wilcoxon sum rank test was used for associations with the processing date, exposure and *PON1* Q192R genotype. Significant associations between principal component 2 and sentrix_ID (BeadChip) and processing date were suggestive for batch effects and were therefore corrected using the ComBat function in the SVA R package [37] (Additional files 2 and 3). Raw and normalized array data were uploaded to the Gene Expression Omnibus (GEO) database and have accession number: GSE90177.

For each sample, the relative cell type contribution was measured using the approach described by Houseman et al. [38]. Reference methylomes of each blood cell type (granulocyte, CD4+ T-cell, CD8+ T-cell, B-cell, monocyte, NK-cell) were obtained from the study of Reinius et al. using the FlowSorted.Blood.450 K R package [39]. The analysis was limited to the 100,000 most variable sites. The top 500 cg-probes associated with the cell types were used to estimate the relative cell type composition in each sample. One-way ANOVA was used to determine differences in relative cell type composition between the exposed and the unexposed children and between children with the QQ and QR/RR genotype. Associations between relative cell type composition and health outcomes (percentage body fat, delta BMI z-scores from birth to school age, and BMI *Z* scores), leptin levels and age were analyzed using simple linear regression.

### Statistical analysis

**Fig. 1:**
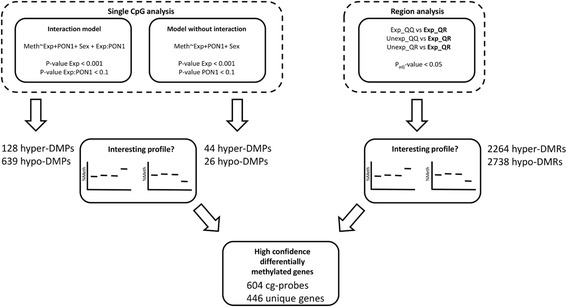
Analysis workflow. Differentially methylated genes were detected using a single CpG and a region-based approach. Only sig-DMPs and sig-DMRs were selected in which the pesticide exposed QR carrier group was either hyper- or hypomethylated in comparison with the other groups (interesting profile). By overlapping the sig-DMPs with the sig-DMRs a high confidence list of differentially methylated genes could be generated

Differential methylation was analyzed both at the single CpG site level and at the region level (Fig. 1). At the single CpG site level, multiple linear regression (Matlab version 2014b, The Mathworks®, Natick, MA, USA) was performed in which methylation was the dependent variable and *PON1* Q192R genotype and prenatal pesticide exposure (yes/no) were the independent variables. Our statistical approach was designed to explain—at the level of methylation—the previously reported gene-environment interaction between the paraoxonase 1 (*PON1*) Q192R genotype and prenatal pesticide exposure leading to an adverse cardio-metabolic risk profile at school age among children carrying the R-allele [19]. Thus, our primary interest was to identify methylation marks associated with exposure that were more altered in R-allele carriers than in QQ-homozygotes. Two statistical models were included in our statistical approach. In the first model, effect modification (interaction) of exposure by *PON1* Q192R genotype was allowed by including main effects (exposure and genotype) and cross-product terms (exposure*genotype) in the models. Statistical significant effects of exposure in the *PON1* 192QR/RR group were defined as follows: *P* value interaction term ≤0.1 and *P* value of exposure in the QR/RR group ≤ 0.001. This model allows studying synergistic effects where the combined effect of prenatal exposure and in the QR/RR group is greater than the sum of the effects of each factor alone. In the second model, effect modification of exposure by *PON1* Q192R genotype was not assumed (no cross product term included). Statistical significant effects of exposure were defined as follows: *P* value of exposure ≤0.001, *P* value of *PON1* genotype ≤ 0.1. In this model the combined effect of exposure and being R-allele carrier is equal to the sum of the effect of each factor separately. For both models, the associations were adjusted for child sex. To identify probes that were most aberrant in the exposed QR/RR group, we set an additional filter for both models in which we defined that the prenatally exposed QR/RR group should either be highest or lowest methylated (based on mean methylation level) as compared to the other three groups (exposed QQ, unexposed QR/RR and unexposed QQ). These sites are defined as significantly differentially methylated positions (sig-DMPs) in the remainder of this text. Sig-DMPs were annotated using the HumanMethylation450 v1.2 manifest file. The freely available EpiExplorer tool was used to add further annotation including chromatin state segmentation and histone modifications based on the UCSC hg19 browser [40]. Genomic locations of transcription factor binding sites (TFBS) were directly downloaded from the UCSC h19 genome browser. Enrichment or depletion of sig-DMPs in a particular genomic region was determined using the Fisher’s exact test.

Differentially methylated regions (DMRs) were detected using the limma-based DMRcate R package [41]. We only looked for regions differentially methylated between the exposed QR/RR group and one of the other groups (exposed QQ, unexposed QR/RR and unexposed QQ). In line with identification of sig-DMPs, significant regions (*P*_adj_ value < 0.05) were selected in which the exposed R-allele carriers showed either the highest or the lowest methylation state which are called sig-DMRs in the remainder of this text. *P* values were corrected for multiple testing using the Benjamini-Hochberg method (*P*_adj_).

### Pyrosequencing

We used bisulfite pyrosequencing to further verify the methylation differences observed in the methylation array. We selected regions in four genes that are known to be involved in metabolism: *LEP*, *GPR39*, *PPARG*, and *OPCML* (Additional file 4). *LEP* DNA methylation has been associated with BMI, birth weight, and cholesterol levels [42–44]. Also, maternal conditions have an effect on the methylation status of the *LEP* promoter [45–48]. GPR39 belongs to the ghrelin receptor family and was shown to be associated with obesity [49]. PPARG is a nuclear receptor involved in regulation of lipid and metabolism as well as a target for some obesogenic endocrine disruptors [20, 50–53]. Furthermore, PPARγ is directly involved in the regulation of PON1 gene expression [54–56]. OPCML (Opioid Binding Protein/Cell Adhesion Molecule-like) is a member of the IgLON family. A SNP in the OPCML gene was associated with coronary artery calcified plaque in African Americans with type 2 diabetes [57]. A mouse and human GWAS analysis identified an OPCML SNP associated with obesity traits and visceral adipose/subcutaneous adipose ratio, respectively [58, 59]. 1 μg DNA from each sample was bisulfite-converted using the EpiTect Fast bisulfite Conversion Kit (Qiagen, Hilden, Germany) according to manufacturer’s instructions. 15 ng of bisulfite-treated DNA was subsequently used in PCR amplification using the PyroMark PCR Kit (Qiagen, Hilden, Germany). Reverse primers were biotinylated to get biotin-labeled PCR products. Finally, DNA sequences were pyrosequenced using the PyroMark Q24 Advanced instrument (Qiagen, Hilden, Germany). First, streptavidin-coated Sepharose beads (High Performance, GE Healthcare, Uppsala, Sweden) were used to immobilize the biotin-labeled PCR products. Subsequently, PCR products were captured by the PyroMark vacuum Q24 workstation, washed and denaturated. The single stranded PCR products were mixed and were annealed with their corresponding sequencing primer. After the pyrosequencing run was finished, the results were analyzed using the PyroMark Q24 Advanced software (Qiagen, Hilden, Germany). Biotinylated-reverse, forward, and sequencing primers were designed using the PyroMark Assay Design 2.0 software (Qiagen, Hilden, Germany) (Additional file 1).

### Mediation analysis

For a subset of sig-DMPs and sig-DMRs we analyzed (1) whether methylation is a mediator between exposure in *PON1* 192R-allele carriers and leptin levels; and (2) whether methylation is a mediator between exposure in *PON1* 192R-allele carriers and body fat accumulation (using delta BMI-score (from birth to school age), and percentage body fat as endpoints). Mediation analysis was restricted to the subset of the methylation data that overlap between the list of sig-DMPs (interaction model) and sig-DMRs. The analysis was performed by the procedure described by Baron and Kenny (1986) [60]. Leptin concentrations were logarithmically (ln) transformed prior to analysis. In mediation analysis considering body fat percentage and leptin, the models were adjusted for sex. As sex was already considered when calculating BMI *Z* score, associations considering mediation between pesticide exposure and BMI *Z* score were not adjusted for sex.

To demonstrate mediation, four requirements must be met: (model 1) the dependent outcome variable (leptin or a body fat measure) should be significantly associated with pesticide exposure (independent variable); (model 2) the DNA methylation mark (mediator) should be significantly associated with pesticide exposure; (model 3) the dependent variable should be significantly associated with the DNA methylation mark; and (model 4) the DNA methylation mark should be a significant predictor of the outcome variable, while controlling for pesticide exposure. The estimated exposure-related change in the outcome variables in model 4 should be less than in model 1 to demonstrate partial mediation, and drop to zero to demonstrate full mediation. A *P* value below 0.05 was used as a cut-off for statistical significance in each of the models.

Functionally relevant mediators, i.e., mediators that have been reported to be involved in development of weight gain/obesity, insulin resistance/diabetes, cardiovascular disease, and/or fetal growth retardation were subjected to further statistical analysis. R-package “mediation” was used to calculate the significance of the causal mediation effect using a bootstrapping approach [61]. It should be noted that the age of the children varied between 6 and 11 years at the follow-up examination where blood was collected. As child age might affect methylation levels, the exposed and unexposed children selected for this study were age-matched within each genotype.

### Functional analysis

Ingenuity Pathway Analysis (IPA, Ingenuity Systems®) was used for biological interpretation. The overlap between sig-DMPs and sig-DMRs was determined and used as input for canonical pathway analysis. A Fisher’s exact test was used to determine whether the gene lists include more genes associated with a given pathway as compared to random chance (*P* value ≤ 0.05).

The DisGeNet platform (http://www.disgenet.org/) was used to screen for gene disease associations [62]. The database (currently) contains 429111 gene disease associations for which the platform provides a reliability score (DisGeNET Score). This score ranges from 0 to 1 and takes into account the number and type of sources (level of curation, organisms), and the number of publications supporting the association (for further details we refer to the DisGeNet website). For this manuscript, we extracted the associations with a score above 0.1. By this criterion, 34180 gene disease associations remain in the database. Associated diseases were mapped to the overlapping list of genes between sig-DMPs and sig-DMRs.

## Results

### Descriptive statistics of the study population

Characteristics, inclusive anthropometric data, for the 48 children (6–11 years of age) are presented in Table 1. In accordance with the findings for the whole cohort [19], birth weights were significantly lower and measures of body composition (abdominal circumference, skin fold thickness), increase in BMI *Z* score from birth to school age (delta BMI *Z* score), diastolic blood pressure, and leptin and insulin concentrations at school age were significantly higher in the exposed *PON1* 192QR/RR group compared with the unexposed QR/RR group. For children with the QQ genotype, none of the variables was significantly affected by prenatal pesticide exposure (*P* > 0.05).

### Prenatal pesticide exposure-induced methylation changes at CpG sites enriched in promoter regions in *PON1* 192R-allele carriers

**Fig. 2:**
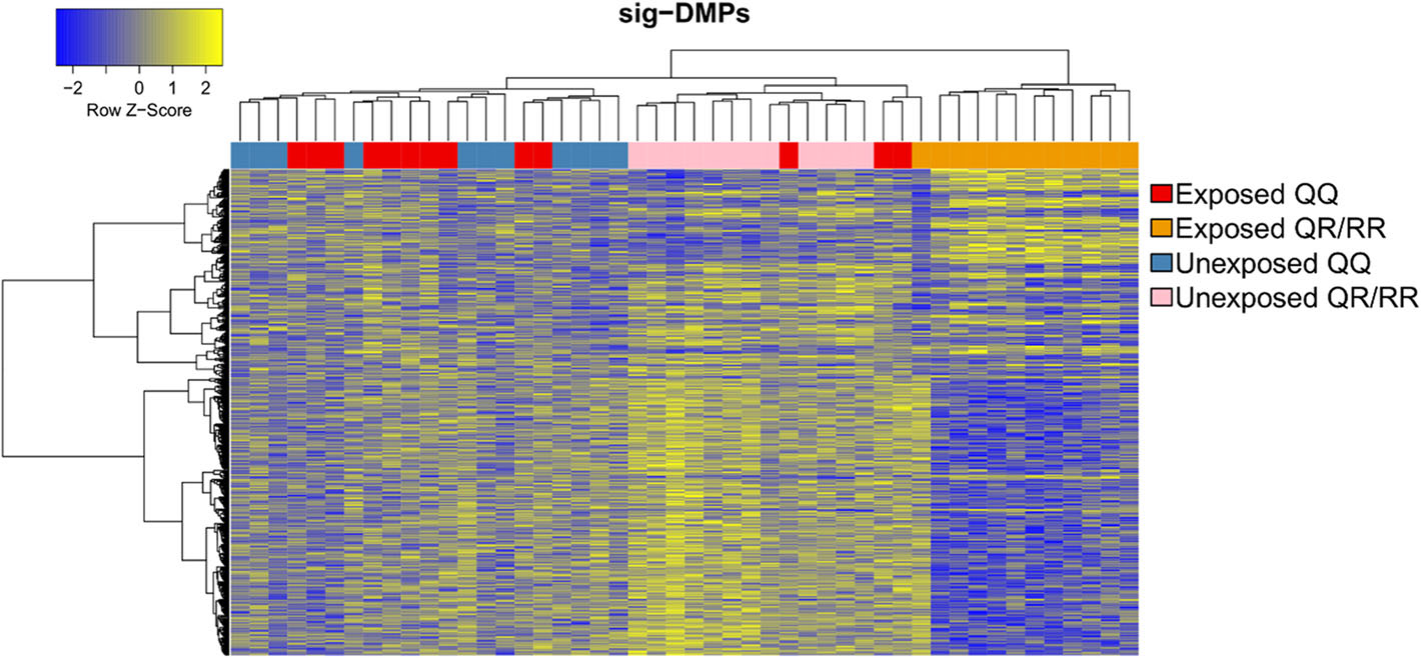
Heatmap clustering representation of the sig-DMPs. Heatmap of the methylation values from the sig-DMPs showing a clear cluster of prenatal pesticide exposed *PON1*-192 R-carrier samples (*orange group*). Hierarchical clustering is based on the euclidean distance and average linkage metric. Higher methylation values are colored in *yellow*, while lower methylation values are colored in *blue*

Genome-wide DNA methylation in whole blood samples from the children was determined by Illumina 450 K methylation arrays and differential methylation patterns related to prenatal pesticide exposure and *PON1* Q192R genotype were analyzed. First differential methylation was detected at the single CpG level using two multiple linear regression models (Fig. 1). Because relative cell type composition was not associated with pesticide exposure and *PON1* Q192R genotype (Additional file 5), differences in cellular composition were not further considered in the workflow of statistical analysis. Allowing effect modification by *PON1* Q192R genotype, 767 sig-DMPs were identified of which 128 were hypermethylated and 639 hypomethylated in prenatally exposed *PON1* 192R allele carriers. When effect modification was not assumed, and the interaction term between exposure and *PON1* genotype was removed from the models, 70 sig-DMPs of which 44 were hypermethylated and 26 hypomethylated in prenatally exposed *PON1* 192R-allele carriers were identified. Hierarchical clustering of the samples using all the sig-DMPs demonstrated a clear cluster of exposed *PON1* 192R-allele carriers (Fig. 2). Confidence in detection of differentially methylated genes was increased by further analysis showing that the changes in methylation were not restricted to single CpGs, but were often located in regions or so called differentially methylated regions (DMRs). 5002 sig-DMRs were identified, of which 2264 were hypermethylated and 2738 hypomethylated in the exposed *PON1* 192R carrier group compared to the other groups. Allowing interaction between exposure and *PON1* Q192R genotype to determine sig-DMPs, 547 out of 767 sites (71.3%) were overlapping with the list of sig-DMRs (Additional file 6). When effect modification was not considered, 57 out of 70 sites (81.4%) were overlapping (Additional file 7).

**Fig. 3:**
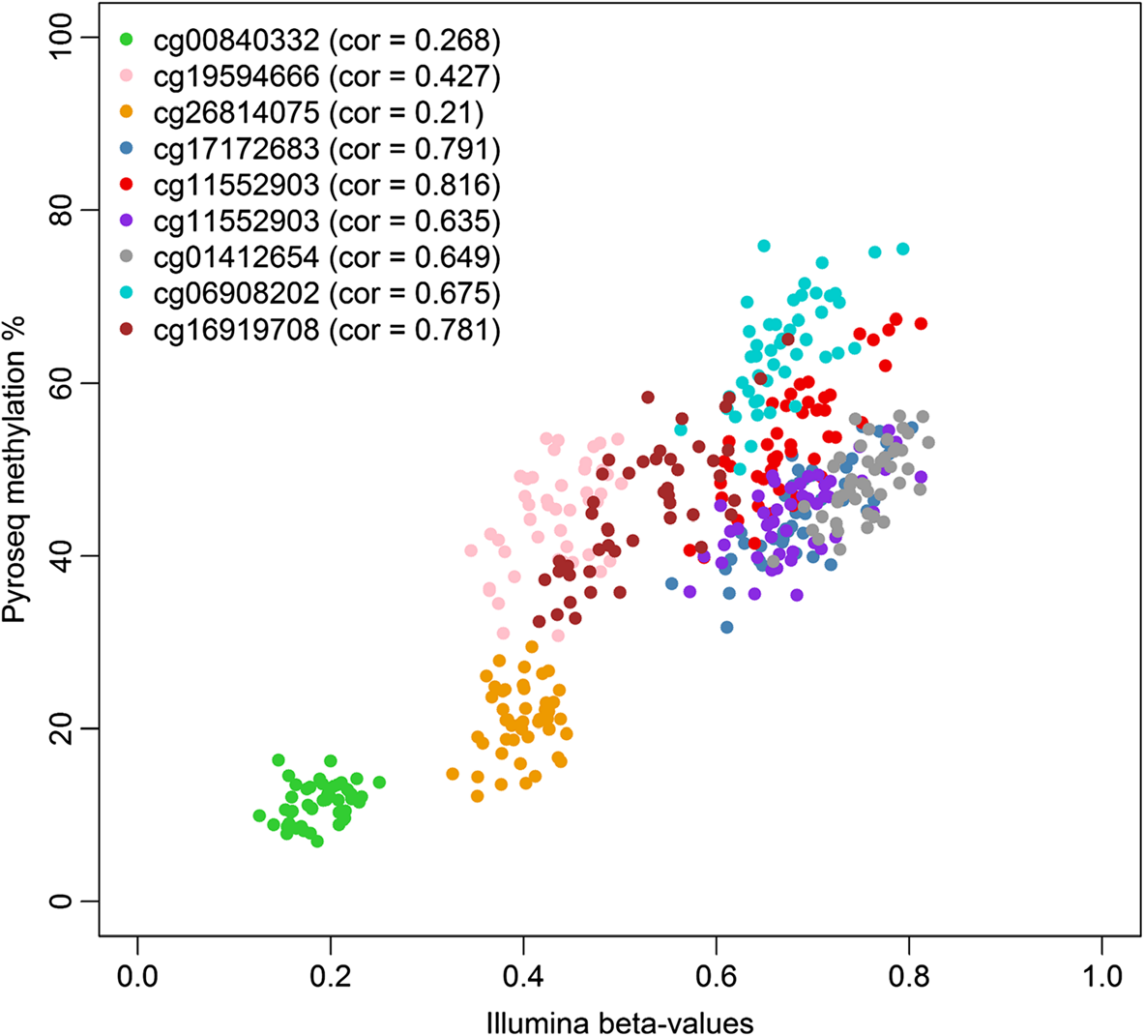
Correlation between the Illumina 450 K beta values and the pyrosequencing methylation percentages. The Pearson’s correlation coefficient for each CpG probe is indicated between brackets. CpG probes cg00840332, cg19594666, and cg26814075 are located in the *LEP* gene, cg17172683, cg11552903, and cg18444763 in the *GPR39* gene, cg01412654 in the *PPARG* gene, and cg06908202, and cg16919708 in the *OPCML* gene

The pyrosequencing methylation percentages confirmed the robustness of Illumina results. They showed significant positive correlations with the Illumina 450 K beta values for all measured CpG probes (Fig. 3), except for two probes in the *LEP* gene (cg00840332 and cg26814075) which were borderline significant (*P* value: 0.07 and 0.16, respectively). The reason for this less strong correlation between the Illumina and the pyrosequencing *LEP* methylation is probably the lower inter-individual methylation variability in this region compared to *GPR39* and *PPARG*.

In accordance with the Illumina results, the pyrosequencing *LEP* methylation values were not associated with pesticide exposure and/or *PON1* Q192R genotype. Furthermore, the serum leptin concentrations were not correlated with *LEP* methylation status (data not shown). For *GPR39*, the region analyzed with pyrosequencing contained three Illumina cg-probes (cg17172683, cg11552903, and cg18444763), which showed a high correlation (*r* > 0.78) between the Illumina beta values and the pyrosequencing methylation percentages. For most CpGs in the pyrosequencing region, we could verify a significant exposure effect, and in each CpG site, prenatally exposed children with the QR/RR genotype had the lowest mean methylation value (Additional files 8 and 9). In the *PPARG* promoter, a region was selected containing one Illumina cg-probe (cg01412654). Also here, the correlation between the 450 K Illumina beta values and the pyrosequencing methylation percentages was strong. However, DNA methylation in this region was not associated with pesticide exposure and/or *PON1* Q192R genotype and did not correlate with PON1 activity (data not shown). A region in the OPCML gene was found to be higher methylated in prenatal pesticide-exposed children carrying the *PON1* 192R-allele. The significant interaction effect between pesticide exposure and *PON1* Q192R genotype could be successfully verified by pyrosequencing. The pyrosequencing methylation values were significantly higher methylated in exposed children compared to unexposed children carrying the *PON1* 192R-allele for most of the CpG sites in the region (Additional file 9).

**Fig. 4:**
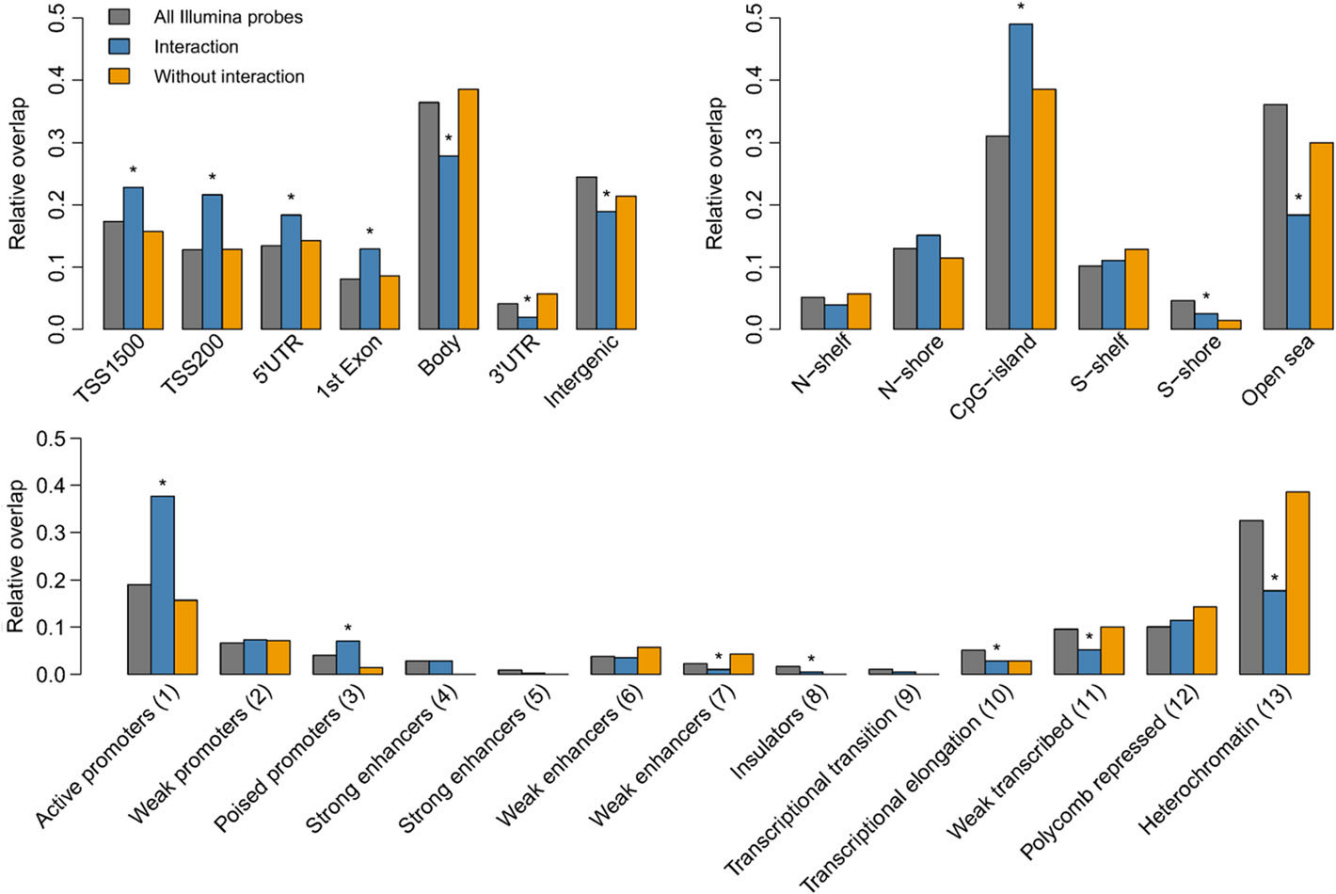
Genomic location of sig-DMPs. DMPs were mapped to gene elements (*top left*), CpG islands (*top right*), and chromatin state segmentations (*bottom*). *Asterisks* indicate significant enrichment or depletion in comparison with all Illumina probes (*gray bars*) measured by the Fisher’s exact test (*P* value < 0.05). Sig–DMPs where the interaction term was significant (*blue bars*) showed an enrichment in promoter regions and CpG islands. Sig-DMPs where no interaction was seen (*orange bars*) showed no significant enrichment or depletion in a particular genomic region

Next, we questioned whether the sig-DMPs were enriched or depleted in a specific genomic location (Fig. 4). Sig-DMPs for which interaction between exposure and *PON1* Q192R genotype was seen, were enriched in promoter regions (200 and 1500 bp upstream of transcription start sites) and depleted in gene bodies, 3′UTRs and intergenic regions. This was also evident when we overlapped the sig-DMPs with different chromatin states, where we observed enrichment in active and poised promoters, while DMPs were depleted in regions like transcriptional elongation, weak transcribed, and heterochromatin regions. Furthermore, DMPs were significantly more located in CpG islands and less observed in CpG poor regions. Sig-DMPs found in the models without an interaction term were not enriched or depleted in a particular genomic region.

We also looked for enrichment in TFBS using available ChIP ENCODE data from the UCSC genome browser. Thirty-nine of the 161 TFBS were significantly enriched for the model with interaction (Bonferroni adjusted *P* value < 0.05) while no enrichment was found for the sig-DMPs found in the model without interaction (Additional file 10).

### DNA methylation differences were enriched for genes involved in neuro-endocrine signaling pathways

Overlapping the list of sig-DMPs with the list of sig-DMRs we obtained a robust and a high confidence list of differentially methylated genes (*N* = 446). This list was used as an input for ingenuity pathway analysis. The top enriched canonical pathways (based on *P* value) were dopamine-DARPP32 feedback cAMP signaling, corticotrophin releasing hormone signaling, nNOS signaling in neurons, CDK5 signaling, and neuregulin signaling (Table 2). In the context of this manuscript, other significantly enriched pathways such as mTOR signaling (rank 9, −log(*P* value) = 1.85) and type II diabetes mellitus signaling (rank 16, −log(*P* value) = 1.51) are also highly relevant.

**Table 2:** Significant enriched Ingenuity canonical pathways

Rank	Ingenuity canonical pathways	−log(*P* value)	Ratio	Hyper-genes	Hypo-genes
1	Dopamine-DARPP32 Feedback in cAMP signaling	3.98	0.07	*CREB5*, *PPP2R2B*, *CACNA1A*	*KCNJ2*, *NOS1*, *GRIN2A*, *GUCY1B3*, *ADCY2*, *PRKCH*, *GNAI3*, *CACNA1D*, *PRKCG*
2	Corticotropin releasing Hormone signaling	2.74	0.07	*CREB5*	*JUND*, *NOS1*, *GUCY1B3*, *ADCY2*, *PRKCH*, *GNAI3*, *PRKCG*
3	nNOS signaling in neurons	2.61	0.11	*CAPN3*	*NOS1*, *GRIN2A*, *PRKCH*, *PRKCG*
4	CDK5 signaling	2.41	0.07	*PPP2R2B*, *CACNA1A*	*CDK5R1*, *NGFR*, *ITGA2*, *LAMB1*, *ADCY2*
5	Neuregulin signaling	2.06	0.07	*EGFR*, *ERBB3*	*CDK5R1*, *ITGA2*, *PRKCH*, *PRKCG*
6	PCP pathway	2.06	0.08		*JUND*, *FZD10*, *RSPO3*, *WNT7B*, *WNT9B*
7	Maturity onset diabetes of young (MODY) signaling	2.03	0.14	*CACNA1A*	*GAPDH*, *CACNA1D*
8	Regulation of eIF4 and p70S6K signaling	2.02	0.05	*PPP2R2B*, *FAU*	*RPS16*, *RPS13*, *RPS10*, *ITGA2*, *IRS1*, *RPS19*
9	mTOR signaling	1.85	0.05	*PPP2R2B*, *FAU*	*RPS16*, *RPS13*, *RPS10*, *IRS1*, *PRKCH*, *RPS19*, *PRKCG*
10	Amyotrophic lateral sclerosis signaling	1.84	0.06	*CAPN3*, *CACNA1A*	*NOS1*, *GRIN2A*, *NEFM*, *CACNA1D*
11	NF-κB activation by viruses	1.8	0.07		*ITGAV*, *CR2*, *ITGA2*, *PRKCH*, *PRKCG*
12	Phosphatidylethanolamine biosynthesis III	1.7	1		*PTDSS2*
13	Role of CHK proteins in cell cycle checkpoint control	1.61	0.07	*PPP2R2B*, *RFC4*	*E2F3*, *CHEK1*
14	Synaptic long-term depression	1.6	0.05	*IGF1R*, *PPP2R2B*	*NOS1*, *GUCY1B3*, *PRKCH*, *GNAI3*, *PRKCG*
15	ErbB signaling	1.53	0.06	*EGFR*, *ERBB3*	*NCK2*, *PRKCH*, *PRKCG*
16	Type II diabetes mellitus signaling	1.51	0.05	*PKM*	*NGFR*, *ADIPOR2*, *IRS1*, *PRKCH*, *PRKCG*
17	G beta gamma signaling	1.49	0.06	*EGFR*	*ADCY2*, *PRKCH*, *GNAI3*, *PRKCG*
18	p70S6K signaling	1.48	0.05	*EGFR*, *PPP2R2B*	*IRS1*, *PRKCH*, *GNAI3*, *PRKCG*
19	Role of osteoblasts, osteoclasts, and chondrocytes in rheumatoid arthritis	1.47	0.04		*FZD10*, *NGFR*, *SMAD5*, *WNT7B*, *ITGA2*, *IL1RAP*, *WNT9B*, *TCF7L2*, *NFATC1*
20	Molecular mechanisms of cancer	1.46	0.04		*RASGRF1*, *ITGA2*, *WNT7B*, *IRS1*, *E2F3*, *GNAI3*, *FZD10*, *SMAD5*, *ADCY2*, *WNT9B*, *PRKCH*, *CHEK1*, *PRKCG*
21	nNOS signaling in skeletal muscle cells	1.45	0.13	*CAPN3*	*NOS1*
22	Factors promoting cardiogenesis in vertebrates	1.42	0.05		*FZD10*, *SMAD5*, *PRKCH*, *TCF7L2*, *PRKCG*
23	RAR activation	1.41	0.04		*REL*, *ERCC2*, *SMAD5*, *NR2F1*, *ADCY2*, *PRKCH*, *RARB*, *PRKCG*
24	Choline degradation I	1.4	0.5	*CHDH*	
25	Sulfate activation for sulfonation	1.4	0.5	*PAPSS2*	
26	Mismatch repair in eukaryotes	1.4	0.13	*RFC4*	*MLH1*
27	Glioma signaling	1.37	0.05	*IGF1R*, *EGFR*	*PRKCH*, *E2F3*, *PRKCG*
28	Netrin signaling	1.36	0.08		*UNC5C*, *NCK2*, *NFATC1*
29	Cellular effects of sildenafil (Viagra)	1.33	0.05	*CACNG6*, *CACNA1A*	*KCNN1*, *GUCY1B3*, *ADCY2*, *CACNA1D*
30	GNRH signaling	1.33	0.05	*EGFR*, *CREB5*	*ADCY2*, *PRKCH*, *GNAI3*, *PRKCG*
31	Protein kinase A signaling	1.31	0.03	*HIST1H1A*, *CREB5*	*PTPN9*, *TIMM50*, *NFATC1*, *GNAI3*, *AKAP12*, *NGFR*, *PTP4A1*, *ADCY2*, *PRKCH*, *TCF7L2*, *PRKCG*
32	Ovarian cancer signaling	1.31	0.05	*EGFR*	*FZD10*, *WNT7B*, *MLH1*, *WNT9B*, *TCF7L2*
33	Colorectal cancer metastasis signaling	1.3	0.04	*EGFR*	*ADRBK1*, *APPL1*, *FZD10*, *WNT7B*, *MLH1*, *ADCY2*, *WNT9B*, *TCF7L2*
34	Agrin interactions at neuromuscular junction	1.3	0.06	*EGFR, ERBB3*	*ITGA2*, *LAMB1*
35	Growth hormone signaling	1.3	0.06	*IGF1R*	*IRS1*, *PRKCH*, *PRKCG*

### DNA methylation (partially) mediates associations between pesticide exposure and higher leptin concentrations, body fat content, and delta BMI *Z* scores

The list of genes that overlaps between sig-DMPs (as identified by the interaction model) and sig-DMRs was also used as input for mediation analysis. We identified, respectively, 20, 31, and 45 candidate methylation marks that (partly) mediate the effect between pesticide exposure and serum leptin concentrations; delta BMI *Z* score; and body fat content. Based on applied cut-off criteria, we were not able to identify methylation marks that mediate the effect on BMI *Z* score. Currently known gene disease associations allowed to extract mediators that were reported to be involved in development of weight gain/obesity, insulin resistance/diabetes, cardiovascular disease, and/or fetal growth retardation. This subset of mediators is given in Table 3. Based on Baron and Kenny’s steps to analyze mediation, the association between pesticide exposure and delta BMI *Z* score was partially mediated by hypomethylation of *UQCRC2*, *MTNR1B* and *GRIN2A*, and by hypermethylation of *FABP4* and *LRP8*. Methylation of *UQCRC2* and *LRP8* was also a partial mediator in the association between pesticide exposure and body fat percentage. *LRP8* was also found to mediate the association between pesticide exposure and serum leptin concentration. The *P* value for significance of the causal mediation effect is included in Table 3 and was below 0.1 for all mediators except for *UQCRC2* and *GRIN2A*. Irrespective of disease association of interest, the full list of potential mediators is provided in Additional file 11 which also includes the outcome of the statistical analysis.

**Table 3:** Methylation marks that partially mediate the association between pesticide exposure and leptin and body fat accumulation in *PON1*-192 R-allele carriers

Outcome	IlmnID	Nearest gene symbol	Gene name	Direction of methylation in exposed R carriers	Diseases	Significance of causal mediation effect (*P* value)
Leptin	cg03366858	*LRP8*	Low density lipoprotein receptor-related protein 8, apolipoprotein e receptor	Hyper	Myocardial infarction (0.22)|nerve degeneration (0.21)|Myocardial infarction, susceptibility to, 1 (finding) (0.2)	0.02
Leptin	cg18202502	*LRP8*	Low density lipoprotein receptor-related protein 8, apolipoprotein e receptor	Hyper	Myocardial infarction (0.22) | nerve degeneration (0.21)|myocardial infarction, susceptibility to, 1 (finding) (0.2)	0.024
Delta BMI *Z* score	cg00810945	*UQCRC2*	Ubiquinol-cytochrome c reductase core protein II	Hypo	Mitochondrial complex iii deficiency, nuclear type 5 (0.41) | obesity (0.21)	0.138
Delta BMI *Z* score	cg06337557	*MTNR1B*	Melatonin receptor 1B	Hypo	Diabetes mellitus, Type 2 (0.26)|polycystic ovary syndrome (0.21) | child development disorders, pervasive (0.21)|acute pancreatitis (0.1)	0.032
Delta BMI *Z* score	cg14152613	*FABP4*	Fatty acid-binding protein 4, adipocyte	Hyper	Carcinoma (0.21)|mammary neoplasms, experimental (0.21) | mammary neoplasms, animal (0.21)|insulin resistance (0.1)|erectile dysfunction (0.1)|diabetes mellitus, experimental (0.1)	0.068
Delta BMI *Z* score	cg15134033	*GRIN2A*	Glutamate receptor, ionotropic, N-methyl D-aspartate 2A	Hypo	Epilepsy (0.21)|colorectal neoplasms (0.21)|epilepsy, rolandic (0.21)|melanoma (0.21)|landau-kleffner syndrome (0.21)|autistic disorder (0.21)|morphine dependence (0.21)|language development disorders (0.21)|epilepsy, focal, with speech disorder and with or without mental retardation (0.21)|speech disorders (0.21)|substance withdrawal syndrome (0.21)|rolandic epilepsy, mental retardation, and speech dyspraxia, autosomal dominant (0.2)|reperfusion injury (0.1)|hypoxia-ischemia, brain (0.1)|sepsis (0.1)|fetal growth retardation (0.1)|central nervous system viral diseases (0.1)|placental insufficiency (0.1)	0.144
Delta BMI *Z* score	cg18202502	*LRP8*	Low density lipoprotein receptor-related protein 8, apolipoprotein e receptor	Hyper	Myocardial infarction (0.22)|nerve degeneration (0.21)|myocardial infarction, susceptibility to, 1 (finding) (0.2)	0.026
Bodyfat	cg00810945	*UQCRC2*	Ubiquinol-cytochrome c reductase core protein II	Hypo	Mitochondrial complex iii deficiency, nuclear type 5 (0.41)|obesity (0.21)	0.174
Bodyfat	cg03366858	*LRP8*	Low density lipoprotein receptor-related protein 8, apolipoprotein e receptor	Hyper	Myocardial infarction (0.22)|nerve degeneration (0.21)|myocardial infarction, susceptibility to, 1 (finding) (0.2)	<0.001
Bodyfat	cg18202502	*LRP8*	Low density lipoprotein receptor-related protein 8, apolipoprotein e receptor	Hyper	Myocardial infarction (0.22)|nerve degeneration (0.21)|myocardial infarction, susceptibility to, 1 (finding) (0.2)	<0.001

Only the subset of genes for which associations with metabolic disease have been reported is listed. DisGeNET Score—indicating reliability of the gene disease associations—is included between brackets

### DNA methylation at the *PON1* promoter is affected by the *PON1*-108CT SNP (rs705379) and negatively correlated with paraoxonase 1 activity

**Fig. 5:**
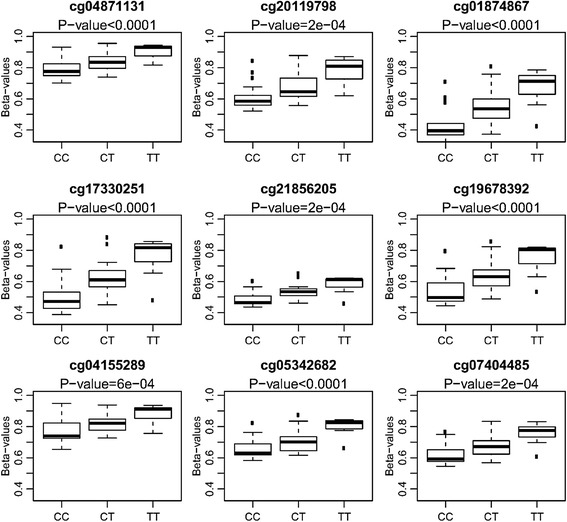
Association between *PON1* methylation and *PON1* C-108T SNP. Individuals homozygous for the T allele showed higher methylation values (beta values) as compared with C-allele carriers. *P* values shown are those from the one-way ANOVA analysis

**Fig. 6:**
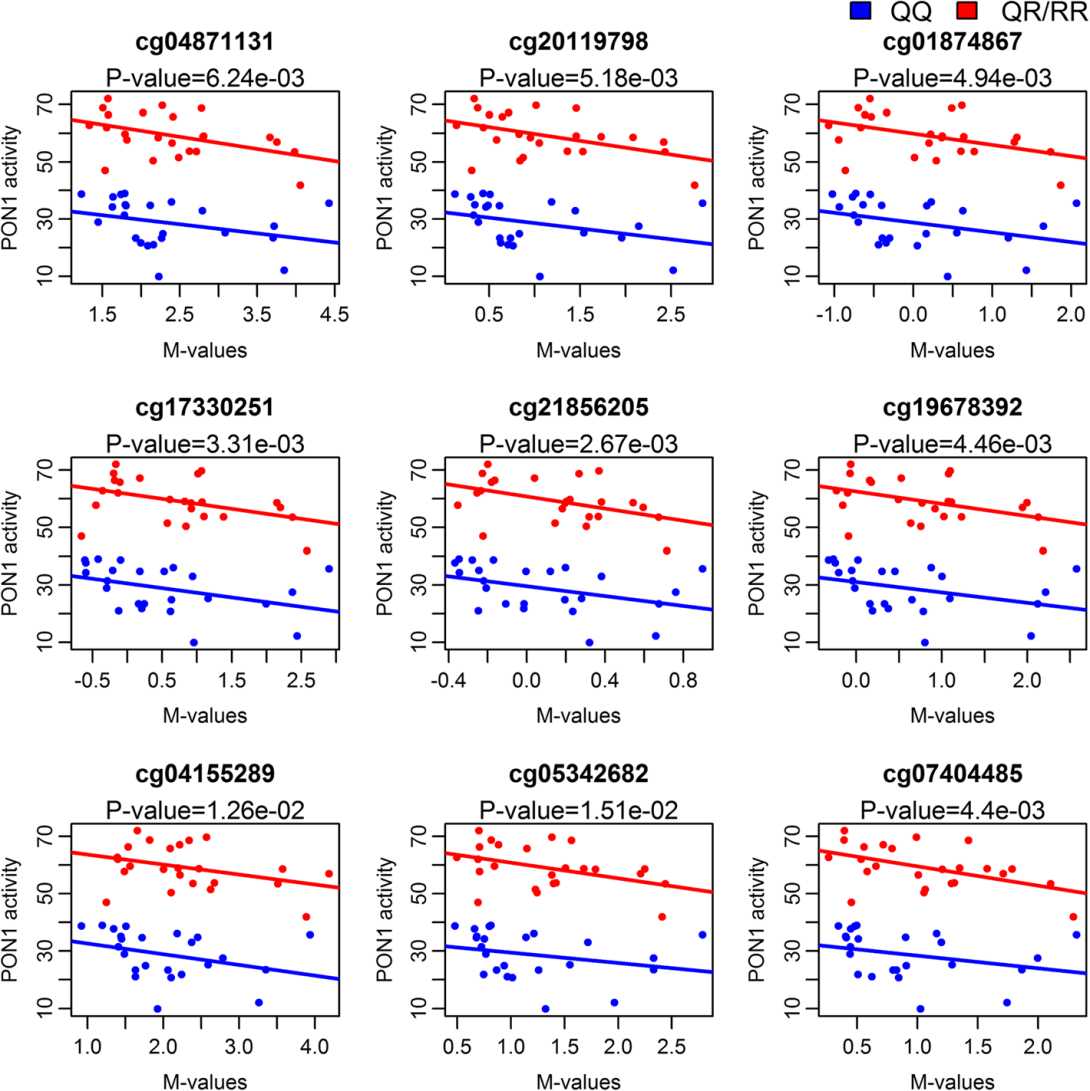
Association between PON1 activity and *PON1* methylation. The *P* values of the main effect for methylation are displayed using the linear model PON1 activity ~ *M* value + *PON1*-192 genotype + sex. *Red* colored samples are *PON1* 192 R-allele carriers, and samples in *blue* are children with the *PON1* 192QQ genotype

Beside the genome-wide DNA methylation effects of the *PON1* Q192R genotype, we also observed a wide variation in DNA methylation in the *PON1* promoter itself for nine Illumina cg-probes. Prenatal pesticide exposure and/or *PON1* Q192R genotype did not affect *PON1* promoter methylation status. However, another polymorphism (rs705379, *PON1* -108CT) in the promoter region of PON1 could explain a large extent of this variation (Fig. 5). Individuals homozygous for the T-allele showed higher methylation values compared with the homozygous C-allele carriers. As expected, heterozygous individuals had an intermediate methylation value. Furthermore, the paraoxonase 1 activity was significantly associated with DNA methylation in the *PON1* promoter region, with higher methylation values resulting in lower paraoxonase 1 activity (Fig. 6). *PON1* Q192R genotype had the strongest effect on PON1 activity, while variation in PON1 promoter methylation led to a smaller but significant effect on PON1 activity.

## Discussion

We found that prenatal pesticide exposure was associated with a differential DNA methylation profile in children carrying the *PON1* 192R-allele compared to children with the *PON1* 192QQ genotype and unexposed children. 767 sig-DMPs were identified of which 128 were hypermethylated and 639 hypomethylated in prenatally exposed *PON1* 192R-allele carriers. The profiles of *PON1* 192R-allele carriers are clustered together. As far as we know, our study is the first one to demonstrate a link between epigenetics and genetic susceptibility towards pesticide exposure in fetal life. Our study supports a linkage of a differential methylation pattern and higher body fat content and serum leptin concentrations in school age children dependent on both *PON1* Q192R genotype and prenatal pesticide exposure.

The majority of the detected sig-DMPs were hypomethylated in exposed children with the *PON1* 192QR/RR genotype. Interestingly, these DMPs were mainly located in gene promoters, CpG islands and transcription factor-binding sites, suggesting a possible direct link with gene expression. To increase the confidence of our findings, we also screened for differentially methylated regions. Most of the single CpG sites were part of a DMR suggesting that these were independent of technical variation and could be considered as reliable.

Technical reliability of the outcomes from the 450 K Illumina methylation array was successfully confirmed by bisulfite pyrosequencing of corresponding CpG probe regions of four selected genes, i.e., *LEP*, *PPARG*, *GPR39*, and *OPCML* for which corresponding probes were available.

*LEP* was chosen because we previously found leptin to be a potential mediator of the association between prenatal pesticide exposure and body fat accumulation in children with the *PON1* 192R-allele [20]. In addition, multiple studies demonstrated associations between *LEP* DNA methylation and BMI, birth weight, and cholesterol concentrations [42–44]. *LEP* was also found to be differentially methylated in the offspring of mothers suffering from the Dutch winter famine [45]. However, our pyrosequencing results did not demonstrate a correlation between leptin DNA methylation and leptin serum concentrations, and prenatal pesticide exposure was not associated with changes in leptin DNA methylation. This suggests that the higher leptin concentration observed in exposed children with the R-allele is not due to a direct effect on DNA methylation of the leptin gene itself. Another gene whose methylation was confirmed by pyrosequencing was *PPARG*, a nuclear receptor controlling the expression of genes involved in lipid storage and glucose metabolism and target for obesogenic compounds [50–53]. Furthermore, PPARγ is involved in the regulation of *PON1* expression [54–56]. However, we did not find a correlation between *PPARG* DNA methylation and PON1 activity (data not shown). In our dataset, prenatal pesticide exposure did not seem to change *PPARG* methylation levels irrespective of *PON1* Q192R genotype.

Reduced *GPR39* DNA methylation observed in prenatally pesticide exposed R-allele carriers was confirmed with pyrosequencing. GPR39 is receptor for obestatin (belonging to the ghrelin receptor family), involved in regulation of appetite and glucose homeostasis [63, 64] and associated with obesity [49]. Furthermore, GPR39 knock-out mice showed an increased fat accumulation due to changes in lipolysis and energy expenditure [49]. So, mis-regulation of this gene due to methylation changes might lead to an obese phenotype. To our knowledge, no other study has yet reported methylation differences in this region associated with obesity or metabolic disorders or showed links with pesticide exposure.

The higher methylation values of the *OPCML* DMR in exposed children carrying the *PON1* 192R-allele could be confirmed by pyrosequencing. *OPCML* encodes for a protein belonging to the IgLON family. OPCML was shown to be a tumor suppressor and inactivated by DNA methylation in a variety of cancer types [65–68]. There is also a link with metabolic diseases, as SNPs in this gene were found to be associated with obesity traits, coronary artery calcified plaque, and visceral adipose/subcutaneous adipose ratio [57–59].

Further analysis revealed that the differences in DNA methylation were most pronounced in genes involved in neuro-endocrine signaling pathways, including “dopamine-DARPP32 feedback in cAMP signaling”, “corticotropin releasing hormone signaling”, “nNOS signaling in neurons”, and “CDK5 signaling”. These pathways are important in the control of food intake and energy balance. Dopamine signaling, for example, is one of the key players in the reward pathway, also controlling food intake and preferences. Reduced dopamine signaling is assumed to induce overeating [69, 70]. In mice, a high-fat diet during pregnancy resulted in altered gene expression and DNA methylation of the dopamine transporter gene in the offspring, leading to an increased preference for sucrose and fat [71]. Another study found similar results in prenatally stressed rats given a high fat-sucrose diet [72]. These studies suggest that prenatal and early life conditions may influence food intake and food preferences later in life through modulation of the dopamine pathway [73–77]. Organophosphate insecticides have been shown to modulate dopamine signaling [78]. Furthermore, low-dose exposure of neonatal rats caused metabolic dysfunction resembling prediabetes, and in adulthood, exposed animals gained excess weight when fed a high fat diet compared to unexposed rats on the same diet [79].

Corticotropin-releasing hormone (CRH) is a neuropeptide secreted in response to stress. However, a role for CRH in regulating energy balance and food intake has also been described [80–82] including a relation to the action of leptin [83].

Also NOS1 neurons are involved in energy balance and food intake [84–86]. Knock-out of *NOS1* in leptin receptor- and NOS1-expressing hypothalamic neurons results in hyperphagic obesity, decreased energy expenditure, and hyperglycemia in mice [85]. Interestingly, organophosphates have been shown to alter NOS1-expressing neurons during development in mice [87, 88].

Neureguline 1 treatment in rodents has been shown to increase serum leptin concentrations, prevent weight gain, and lower food intake. Hence, affecting this pathway may also change food intake and energy metabolism [89, 90].

A limitation of this study is that the methylation profile is measured at the same time as health outcomes and causality as such cannot be proven. Some of the genes that relate to the sig-DMPs are involved in neuro-endocrine pathways that regulate appetite and energy balance, but this study cannot rule out if these sig-DMPs are a consequence of alterations of food habits and physical activity among the exposed children with the *PON1* 192R-allele or an underlying mechanism. However, the mediation analysis suggested that some of the differentially methylated marks are on the mechanistic pathway between prenatal pesticide exposure and the measured outcomes. This result suggests that, at least in some, CpG sites a change in methylation might contribute to metabolic disturbances later in life. Furthermore, the association was not significant between pesticide exposure and BMI *Z* score as such, but between pesticide exposure and delta BMI *Z* score which integrates fat accumulation from birth and onwards to school age.

Interestingly, some of the mediator marks could be linked to specific genes that were reported earlier to play a role in the development of weight gain/obesity, insulin resistance/diabetes, cardiovascular disease, and/or fetal growth retardation: *UQCRC2*, *MTNR1B*, *GRIN2A*, *FABP4*, and *LRP8. FABP4* encodes for a member of the fatty acid-binding protein family regulating lipid trafficking, signaling, and metabolism. Different studies have demonstrated the role of this protein in obesity, type 2 diabetes and atherosclerosis development [91–93]. In ApoE deficient mice with hyperhomocysteine *FABP4* DNA methylation is reduced in the aorta compared to wild type mice, leading to a higher gene expression [94, 95]. *UQCRC2* encodes a protein which is part of the ubiquinol-cytochrome c reductase complex in the mitochondria. UQCRC2 was shown to be downregulated in individuals who were susceptible to weight gain and obesity development [96]. The melatonin receptor 1B (MTNR1B) has a main function in regulating circadian rhythm. Interestingly, several polymorphisms in the *MTNR1B* gene are associated with type 2 diabetes, fasting glucose concentration, and insulin secretion [97–99]. *GRIN2A* encodes for a NMDA glutamate receptor subunit. Polymorphisms in the *GRIN2A* gene are associated with epilepsy and different neurological and mental disorders [100–104]. A decreased gene expression of GRIN2A in rats after intrauterine growth retardation suggests a possible role for this gene in fetal growth and development [105]. *LRP8* encodes for a member of the LDL receptor family. Common polymorphisms in the *LRP8* gene are associated with coronary artery disease, myocardial infarction, and high birth weight [106–110]. Thus, the mediation analysis suggests a mechanistic role of epigenetics in the development of an adverse metabolic risk profile among the prenatally exposed children with the *PON1* R-allele as previously reported for these children [19] and confirmed in the selected subset of children.

A few studies have investigated associations between PON1 genotype and metabolic disturbances in children. A recent study showed a higher risk of insulin resistance (HOMA-IR) in Mexican children with the RR-genotype as compared to children with the QQ or QR genotypes although BMI did not differ between the groups [111]. Among Mexican-American children from an agricultural community in California, a trend of increased BMI *Z* scores with increased number of PON1 192Q alleles was seen [112]. However, potential interactions between PON1 genotype and prenatal exposure to pesticides, or other environmental contaminants, were not investigated in these studies. In our cohort, unexposed QQ-homozygote children also tended to have higher body fat content than unexposed R-carriers, but prenatally pesticide exposed children with the R-allele accumulated more fat during childhood and had a more unhealthy metabolic risk profile at school age than unexposed children and exposed children with the QQ genotype [19].

We also demonstrated that methylation in the *PON1* promoter itself is affected by a SNP (*PON1* -108CT, rs705379). In addition, *PON1* methylation values were negatively associated with paraoxonase 1 activity. These results are in agreement with the outcome of a recent study from Huen and colleagues [113]. They found methylation in the same nine CpG sites to be associated with the *PON1* -108CT polymorphism and also reported an inverse association with AREase activity as a measure of *PON1* expression, both in newborns and 9-year-old children. Furthermore, they demonstrated that *PON1* methylation mediates the relationship between *PON1* expression and the promoter -108 genotype. However, the effect of prenatal pesticide exposure on the health outcomes shown in Table 1 was not modulated by PON1 -108CT genotype (data not shown).

Our findings indicate that the higher vulnerability among children with the R-allele towards prenatal pesticide exposure might be mediated by genotype-specific epigenetic alterations. However, a limitation of this study is that we cannot identify individual pesticides related to these findings, since the study design did not allow bio-monitoring of pesticide exposure in the mothers, and the exposure classification of the mothers encompassed more than 100 pesticides used in different mixtures [11].

However, the existence of mixed exposure is a real-world situation, and the longitudinal design, the blinded exposure classification, and the blinded clinical examinations, and genotyping minimized the possible impact of exposure misclassification and bias.

Since PON1 is known to detoxify some organophosphate insecticides (e.g., chlorpyrifos), and these substances were frequently applied in the mothers’ working areas, organophosphate insecticides could be assumed to be responsible for the observed effects. However, the mechanism is unclear and does not seem to be related to the hydrolysis efficiency, since R-carriers have higher paraoxonase activity than QQ homozygotes. Besides, at relatively low exposure levels, as in this study, the capacity to detoxify organophosphates is considered to be independent of the *PON1* Q192R genotype [114], and furthermore, serum PON1 activity was reported to be low in newborns and may be even lower before birth, as indicated by lower activity in premature compared to term babies [115, 116]. Thus, differences in fetal detoxification of pesticides related to *PON1* genotype might not be a likely explanation of the exposure-related difference in methylation pattern between children with the QR/RR and QQ genotype.

Another limitation of the study is that DNA methylation analyses were performed in white blood cells as surrogates for the target tissues. We do not know whether the differences in DNA methylation patterns found in blood mirror a similar change in adipose tissue, for example. A recent study from Huang et al. demonstrated several potential limitations in using methylation profiles in blood to mirror the corresponding profile in target tissues by comparing paired blood and adipose tissue methylation profiles [117]. Furthermore, the composition of blood cell types may be variable and might affect the DNA methylation analyses. In our dataset, prenatal pesticide exposure and/or *PON1* Q192R genotype did not affect the relative blood cell counts determined by the reference-based method of Houseman. Cell counts were not included in the models due to the small sample size of the study. Since we found that some of the health effects (mainly leptin) were associated with cell type count (Additional file 12), we cannot exclude that the results of the mediation analysis were biased by differences in cell type composition. Based on the data of Reinius et al. [39], methylation of only two CpG probes (cg18202502 and cg15134033) in Table 3 were slightly associated with cell types (data not shown). Methylation in the other CpG probes in Table 3 was not significantly different between the blood cell types.

Finally, the small number of subjects included in this exploratory study is a clear weakness because of the limited statistical power. Despite these constraints, our findings suggest that DNA methylation might be a link between prenatal pesticide exposure and cardio-metabolic risk profile in children carrying the PON1 192R-allele. The findings deserve further investigation in a larger study with quantitative data on pesticide exposure. Whether this DNA methylation pattern is unique to pesticide exposure or is shared by other adverse prenatal environmental factors also needs further investigation.

## Conclusions

In summary, our data indicate that DNA methylation may be an underlying mechanism explaining an adverse cardio-metabolic risk profile in prenatally pesticide-exposed children carrying the *PON1* 192R-allele.

## Additional files

Additional file 1: Primer sequences. (XLSX 10 kb)

Additional file 2: PCA before and after batch effect correction for Sentrix_ID and processing date using ComBat. (TIFF 240 kb)

Additional file 3: Associations between the first eight principal components and covariates before and after ComBat batch correction. Associations between principal components and Sentrix_ID were measured using the Kruskal-Wallis test. Associations between principal components and processing date, exposure and *PON1* Q192R genotype were measured using the two-sided Wilcoxon sum rank test. (TIFF 102 kb)

Additional file 4: Genomic location of the pyrosequencing assays represented as a UCSC genome browser track. The first track indicates the sequence analyzed by pyrosequencing (Seq_to_analyse). Other custom tracks include: CpG islands, Dnase I hypersensitivity clusters, H3K27ac histone marks, transcription factor-binding sites, and the Illumina 450 K methylation probes. A) *LEP* assay B) *GPR39* assay C) *PPARG* assay and D) *OPCML* assay. (TIFF 3014 kb)

Additional file 5: Relative cell type contribution estimated by the Houseman approach. Differences in cell type composition between the exposure groups were measured using one-way ANOVA. (TIFF 259 kb)

Additional file 6: Sig-DMPs overlapping with DMRs (interaction model). For each DMP *P* values are given for the interaction between pesticide exposure and *PON1* genotype (P.Value.int_EXP:PON1), and for the exposure effect PON1 R-allele carrier group (P.Value.EXP_when PON1 QR/RR). The mean beta values in each exposure group are listed. (XLSX 82 kb)

Additional file 7: Sig-DMPs overlapping with DMRs (model without interaction). For each DMP *P* values are given for the *PON1* effect (P.Value.PON1) and exposure effect (P.Value.EXP). The mean beta values in each exposure group are listed. (XLSX 16 kb)

Additional file 8: Outcome of GPR39 DMR pyrosequencing. *Boxplots* showing methylation differences between the exposure groups in the *GPR39* pyrosequencing region. *P* values shown are those of the exposure effect. (TIFF 193 kb)

Additional file 9: Outcome of GPR39 DMR pyrosequencing. (XLSX 11 kb)

Additional file 10: Enrichment of TFBS for DMPs significant in the interaction model. *P* value from the Fisher’s exact test were adjusted using the Bonferroni correction. (XLSX 10 kb)

Additional file 11: Mediation analysis. Outcome statistics and gene disease associations of (partial) mediators between pesticide exposure and body fat measures in *PON1* R-allele carriers. (XLSX 47 kb)

Additional file 12: Association between estimated blood cell counts and health outcomes. Simple linear regression was used to determine associations between the relative blood cell type composition and the health outcomes (body fat, BMI *Z* score, delta BMI *Z* score, leptin levels, and age). (XLSX 11 kb)

## Abbreviations

DMP: Differentially methylated posistion
DMR: Differentially methylated region
IPA: Ingenuity Pathway Analysis
PAI-1: Plasminogen activator inhibitor type-1
PON1: Paraoxonase 1
TFBS: Transcription factor binding site
