# Supplementary figures and images for: Interaction between prenatal pesticide exposure and a common polymorphism in the PON1 gene on DNA methylation in genes associated with cardio-metabolic disease risk—an exploratory study
Source: Clin Epigenetics. 2017 Apr 5;9:35. doi: 10.1186/s13148-017-0336-4 (PMC5382380; doi:10.1186/s13148-017-0336-4)

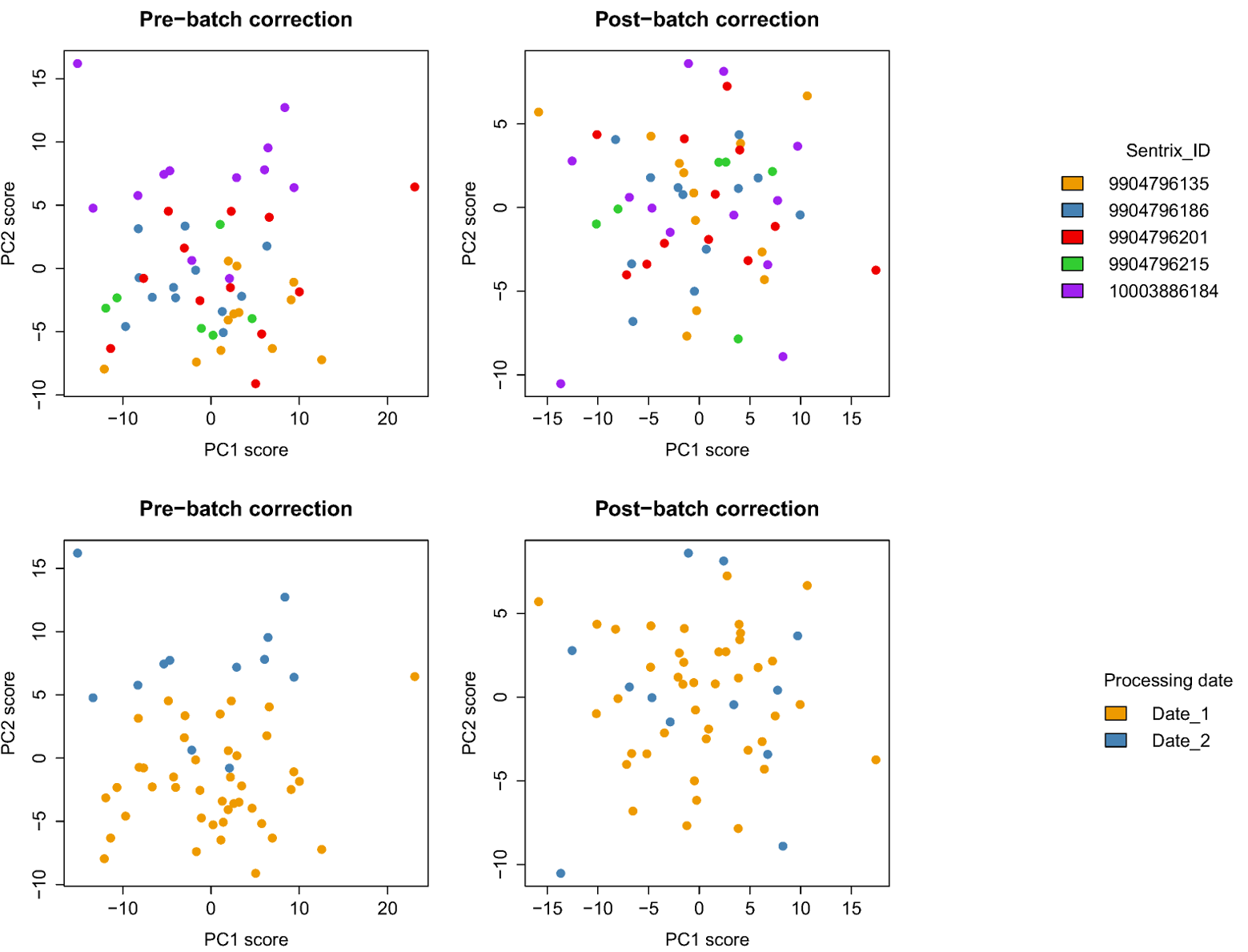

Supplement: Supplementary file 2 — PCA before and after batch effect correction for Sentrix_ID and processing date using ComBat. (TIFF 240 kb) [file 13148_2017_336_MOESM2_ESM.tiff]

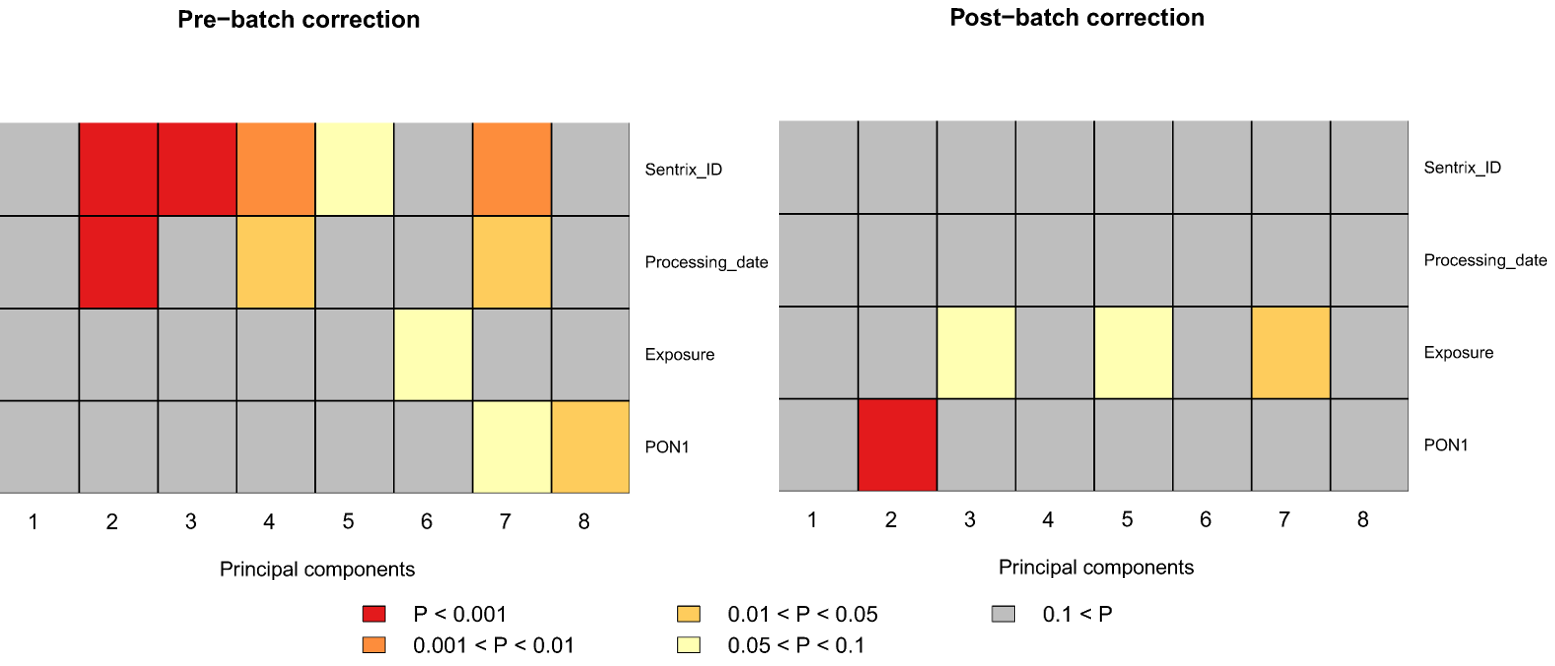

Supplement: Supplementary file 3 — Associations between the first eight principal components and covariates before and after ComBat batch correction. Associations between principal components and Sentrix_ID were measured using the Kruskal-Wallis test. Associations between principal components and processing date, exposure and PON1 Q192R genotype were measured using the two-sided Wilcoxon sum rank test. (TIFF 102 kb) [file 13148_2017_336_MOESM3_ESM.tiff]

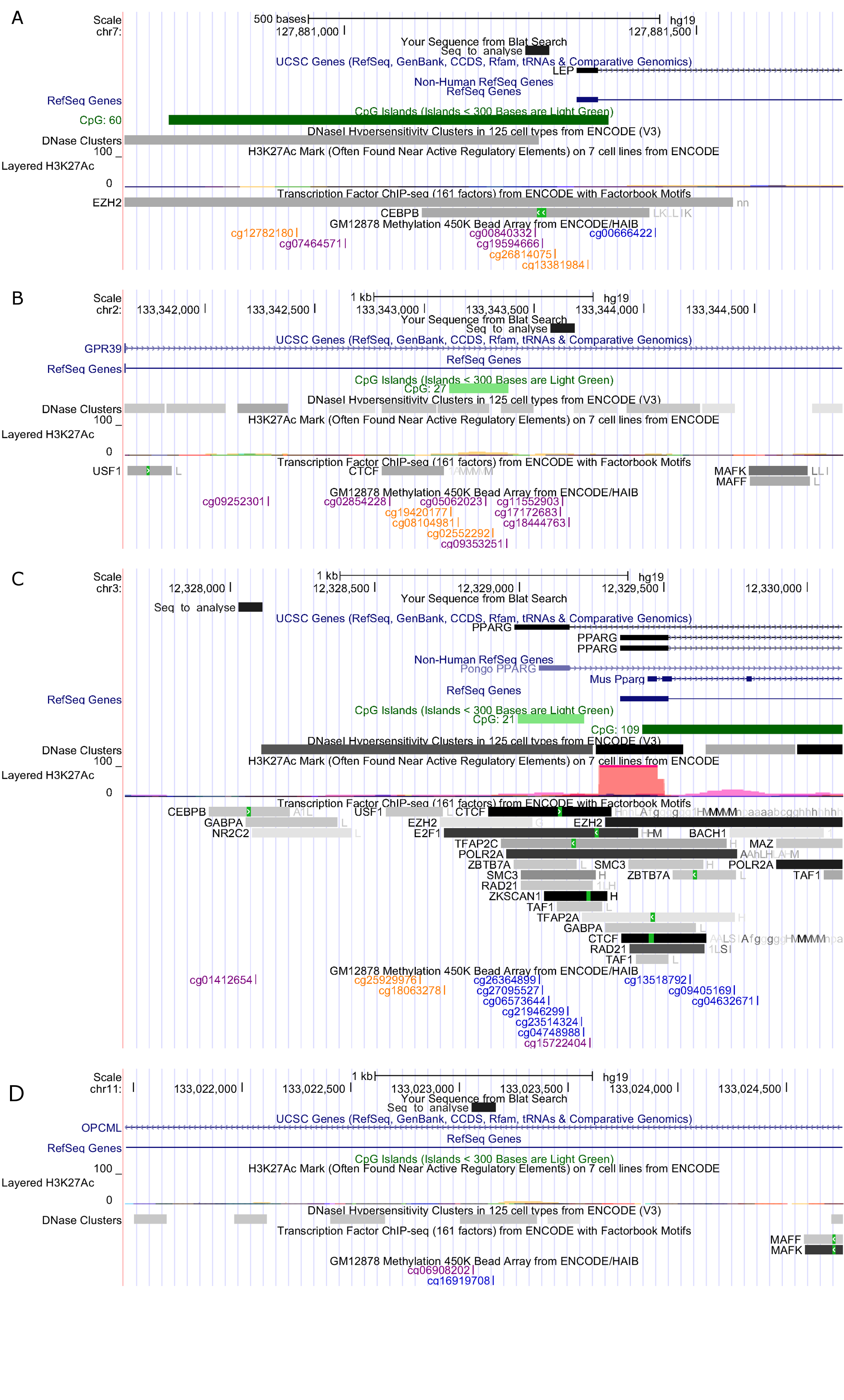

Supplement: Supplementary file 4 — Genomic location of the pyrosequencing assays represented as a UCSC genome browser track. The first track indicates the sequence analyzed by pyrosequencing (Seq_to_analyse). Other custom tracks include: CpG islands, Dnase I hypersensitivity clusters, H3K27ac histone marks, transcription factor-binding sites, and the Illumina 450 K methylation probes. A) LEP assay B) GPR39 assay C) PPARG assay and D) OPCML assay. (TIFF 3014 kb) [file 13148_2017_336_MOESM4_ESM.tiff]

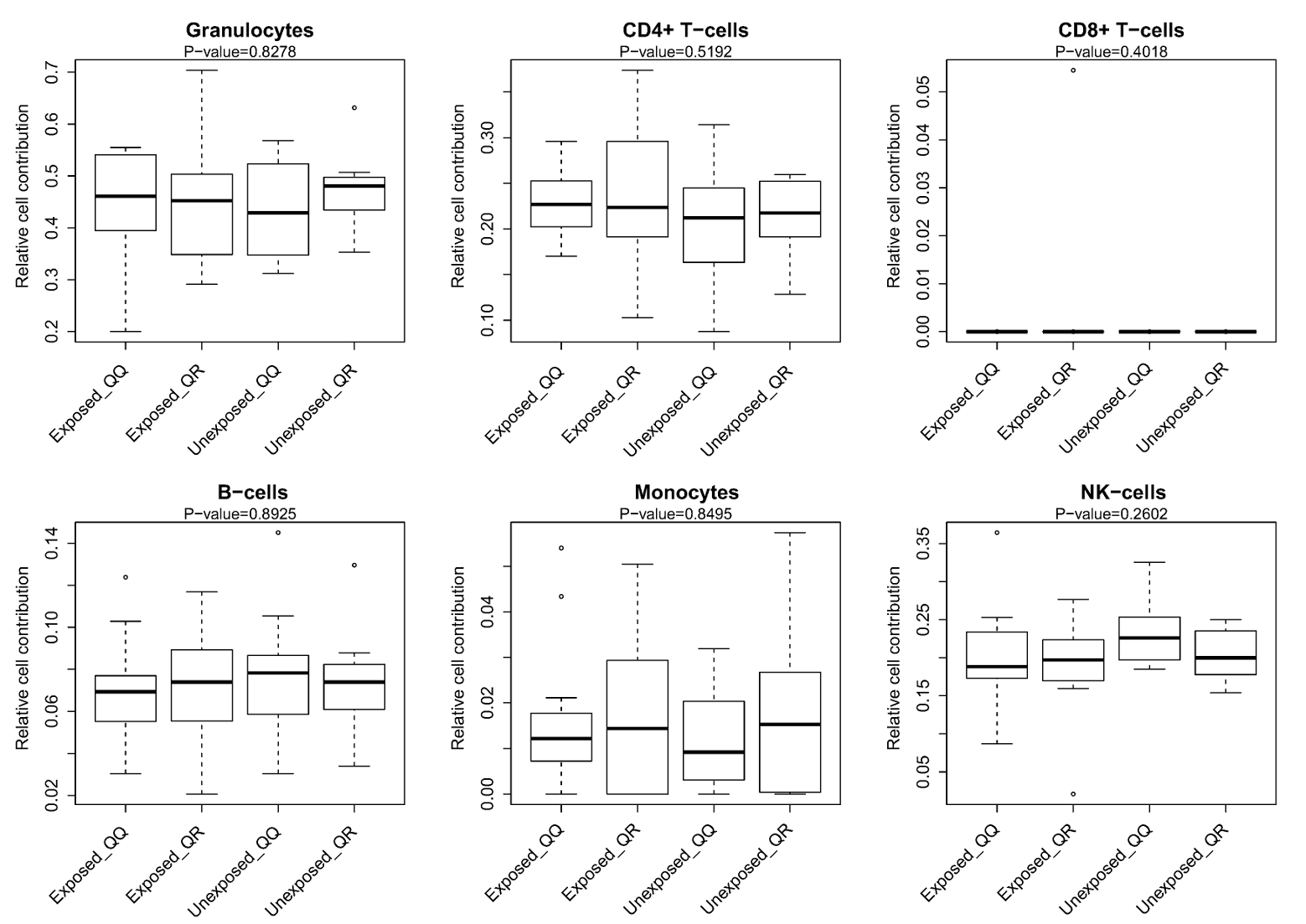

Supplement: Supplementary file 5 — Relative cell type contribution estimated by the Houseman approach. Differences in cell type composition between the exposure groups were measured using one-way ANOVA. (TIFF 259 kb) [file 13148_2017_336_MOESM5_ESM.tiff]

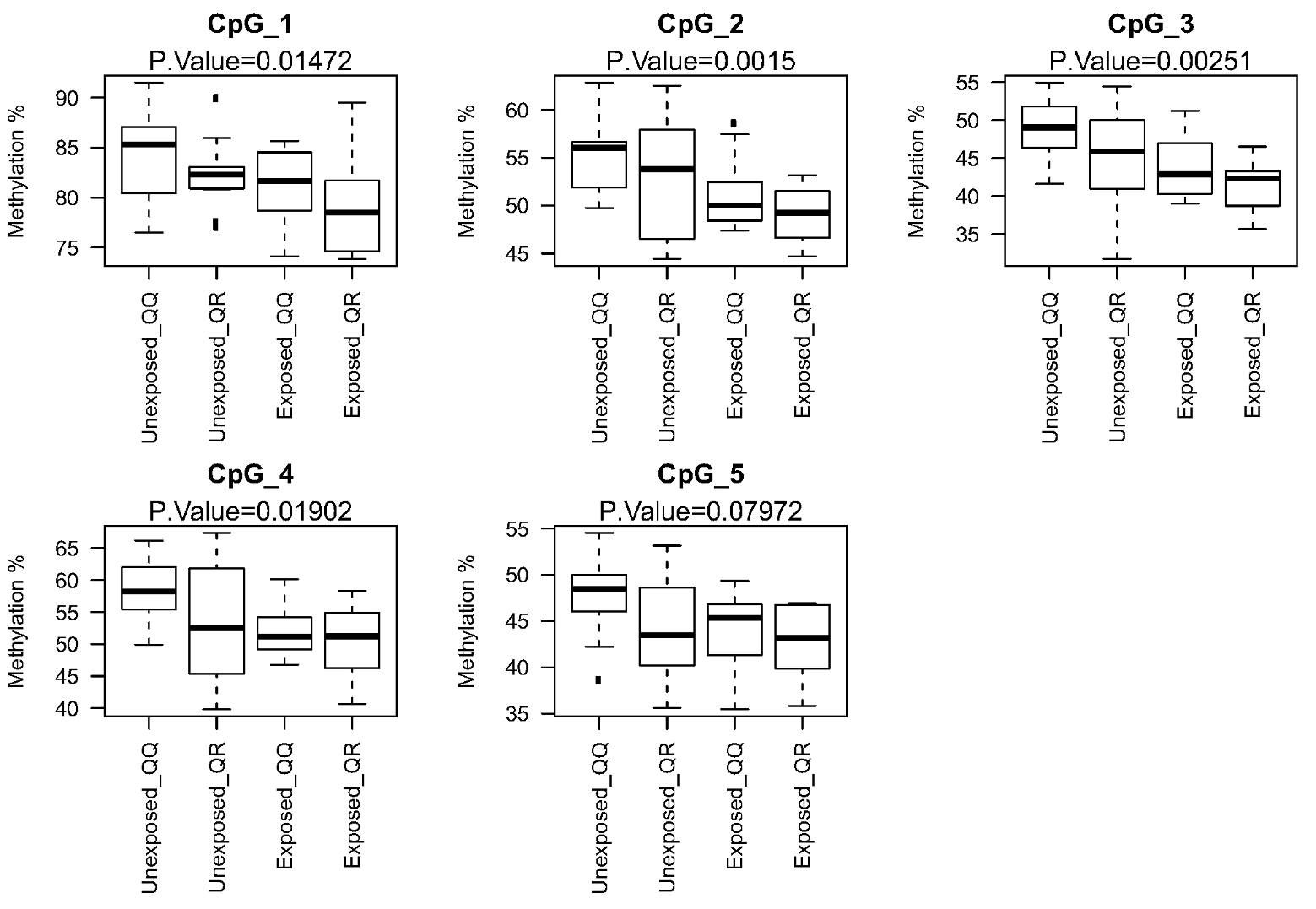

Supplement: Supplementary file 8 — Outcome of GPR39 DMR pyrosequencing. Boxplots showing methylation differences between the exposure groups in the GPR39 pyrosequencing region. P values shown are those of the exposure effect. (TIFF 193 kb) [file 13148_2017_336_MOESM8_ESM.tiff]
